# Caffeine intake and anxiety: a meta-analysis

**DOI:** 10.3389/fpsyg.2024.1270246

**Published:** 2024-02-01

**Authors:** Chen Liu, Licheng Wang, Chi Zhang, Ziyi Hu, Jiayi Tang, Junxian Xue, Wenchun Lu

**Affiliations:** ^1^School of International Education, Xuzhou Medical University, Xuzhou, China; ^2^School of Public Health, Xuzhou Medical University, Xuzhou, China; ^3^School of Management, Xuzhou Medical University, Xuzhou, China

**Keywords:** coffee, caffeine, anxiety, meta-analysis, healthy population

## Abstract

The results from studies on relationship between caffeine intake and risk of anxiety remains controversial, so we conducted a meta-analysis to summarize the evidence about the association between caffeine intake and risk of anxiety. Relevant articles were identified by researching PubMed, Web of Science, Cochrane library, Embase, CNKI, WANFANG DATA, SinoMed and VIP from the inception to December, 2022. Three investigators independently sifted through the literature, extracted the data, and evaluated the quality of the included studies based on predetermined selection criteria and assessed articles with Risk of bias assessment tool for Cochrane systematic reviews and analytical cross-sectional study quality assessment tool from JBI PACES. After assessing the quality of the literature, meta-analysis was performed using Revman 5.4 and Stata 12.0. Data were obtained from eight articles, and 546 participants from 14 studies in eight articles from healthy populations were included in the caffeine-anxiety analyses. As the scales used to assess anxiety vary in the literature, we chose standardized mean difference as the outcome indicator. In terms of overall effect, the results of the meta-analysis showed that caffeine intake increased the risk of anxiety [SMD = 0.94, 95% Cl = (0.28, 1.60), *p* < 0.05]. After suspecting that dose size might be responsible for the heterogeneity by sensitivity analysis, we performed subgroup analysis according to dose size and found that low-dose caffeine intake moderately increased the risk of anxiety [SMD = 0.61, 95%Cl = (0.42, 0.79), *p* < 0.05], whereas high-dose caffeine intake had a highly significant increase in the risk of anxiety [SMD = 2.86, 95%Cl = (2.50, 3.22), *p* < 0.05]. The results confirm that caffeine intake is associated with an elevated risk of anxiety in healthy individuals without psychiatric disorders, especially when the intake dose is greater than 400 mg.

## Introduction

Coffee is one of the best-selling drinks in the world which has a powerful impact on long-term health. According to the Washington Post (2015), the world consumes 2 billion cups of coffee every day. Coffee consumption in China is also increasing year by year. Coffee consists of many bioactive substances with potential antioxidant, anti-inflammatory or anti-cancer effects such as chlorogenic acid, caffeine, alkaloids, caffeol (Cano-Marquina et al., 2013; Ludwig et al., 2014), among which caffeine is classified as a central nervous system (CNS) stimulant and an organic molecule known as methylxanthine.

Caffeine has three different mechanisms of action on the central nervous system to produce a psychostimulant effect, one of which involves the stimulatory antagonism of methylxanthines at the level of adenosine receptors. Four receptors, A1, A2A, A2B, and A3, comprise the adenosine system, of which A1 and A2A bind to caffeine with high affinity and in a reversible manner at normal physiologic doses. Adenosine receptors are mainly located in the hippocampus, amygdala and prefrontal cortex. Adenosine A1 and A2A receptors act as neuromodulators that modulate the activity of other neurotransmitters such as glutamate, gamma-aminobutyric acid (GABA), acetylcholine, 5-hydroxytryptophan, and dopamine (Fredholm et al., 2005), which have been implicated in anxiety. A1 and A2A receptors are also involved in physiological mechanisms such as vasoconstriction and microglial cell functioning (Fredholm et al., 2005) as well as the modulation of a variety of psychological functions including sleep, arousal, memory and anxiety (Ribeiro et al., 2002; Wei et al., 2014). In summary, studies have shown that adenosine receptor blockade can cause anxiety (Maximino et al., 2011), and that caffeine, a methylxanthine substance, is an antagonist of adenosine receptors, which increases energy metabolism in the brain but at the same time reduces cerebral blood flow, leading to relative hypoperfusion, activation of norepinephrine neurons, and effects on the local release of dopamine, leading to anxiety (Nehlig et al., 1992).

According to the World Health Organization (WHO), the global prevalence of anxiety disorders has increased by 15 percent since 2005, with nearly 264 million people suffering from anxiety disorders in 2015 (World Health Organization, 2017). In addition, an estimated 25% increase in the global prevalence of anxiety disorders was recently reported during the 2019 coronavirus disease (COVID-19) pandemic (COVID-19 Mental Disorders Collaborators, 2021). Home confinement during an epidemic has an impact on the population, including changes in sleeping habits and eating habits. People consume unhealthy foods and develop unhealthy habits, including increased caffeine intake, while anxiety and stress levels are among the most reported in studies of physical and mental health (Ammar et al., 2020; Taheri et al., 2023). People with anxiety disorders often experience intense and excessive fear and worry, and these feelings are often accompanied by physical tension and other behavioral and cognitive symptoms that are difficult and distressing to control. If left untreated, they can last for a long time. Anxiety disorders interfere with daily activities and can jeopardize a person’s family, social, school or work life, so research into the relationship between caffeine and anxiety has important public health implications.

Several epidemiologic studies have found a link between caffeine and anxiety, but the results of the existing literature are inconsistent. For example: In a cross-sectional analysis conducted in Iran to examine the association between caffeine intake and symptoms of psychological disorders in adults, the trial demonstrated a significantly lower probability of experiencing anxiety symptoms with weekly or more coffee consumption compared with no coffee consumption, consistent with the findings of a prospective cohort study on the association between tea consumption and anxiety symptoms conducted in Singapore (Chan et al., 2018; Nouri-Majd et al., 2022), while opposite conclusions were reached in a cross-sectional study investigating whether there is a correlation between caffeine intake and anxiety among college students at Florida State University, a randomized controlled trial of the effects of caffeine on mood performance among college student volunteers at the University of Bristol, and a cohort study using the resources of a UK Biobank (Smith et al., 1999; Bertasi et al., 2021; Min et al., 2023). As previous individual studies may not have been of sufficient quality to obtain reliable data, there is still a lack of meta-analysis between caffeine intake and anxiety in healthy populations, although there have been some meta-analysis on the effects of caffeine and anxiety episodes in patients with panic attacks and on the relationship between caffeine intake and symptoms of depression (Klevebrant and Frick, 2022; Torabynasab et al., 2023).

Controversy exists regarding the relationship between caffeine intake and the risk of anxiety, and in order to elucidate the relationship between caffeine and anxiety in healthy populations, meta-analysis of the literature on caffeine intake and anxiety that met predetermined inclusion and exclusion criteria were selected for this study to provide evidence-based evidence for anxiety prevention.

## Methods

### Search strategy

This study performs meta-analysis and reports findings in accordance with Systematic Reviews and Meta-analysis (PRISMA) Statement (Liberati et al., 2009). We used computer searches of PubMed, Web of Science, Cochrane library, Embase, CNKI, WANFANG DATA, SinoMed and VIP databases to retrieve qualified randomized controlled studies and observational studies on caffeine and anxiety. The computer search included keywords such as “coffee,” “caffeine” and “anxiety,” and the search format was a combination of thematic and free-form terms. PubMed database search formula: (“Coffee” [MeSH Terms] OR (“Caffeine” [MeSH Terms] OR “Vivarin” [Title/Abstract])) AND (“Anxiety” [MeSH Terms] OR (“Angst” [Title/Abstract] OR “Nervousness” [Title/Abstract] OR “Anxiousness” [Title/Abstract]) OR (“Anxiety Disorders” [MeSH Terms])). The search strategy was customized slightly for different databases. We searched for studies and related papers published before December 1, 2022.

### Literature inclusion and exclusion criteria

Studies were included in this meta-analysis if they met the following criteria: (1) the included studies were randomized clinical trial, prospective cohort studies, case–control studies and cross-sectional studies; (2) the study population was healthy population without psychiatric disorders; (3) the intervention was caffeine consumption. The trial group received total coffee or caffeinated beverage and the control group received decaffeinated coffee or placebo and (4) the mean and standard deviation of anxiety scale scores were used as outcome indicators.

Studies were excluded if (1) subjects already suffered from anxiety or were in a specific stressful situation; (2) presence of other psychotropic drug interventions; (3) republished studies with the most detailed data were selected; (4) a study in which data were ambiguous, incomplete, or unable to be transformed or merged; (5) lack of original data.

### Literature and data extraction

The systematic literature search was performed following the PRISMA guidelines (Moher et al., 2009). Two reviewers sifted through the study independently, and any disagreement was resolved by discussion between the two reviewers. If consensus could not be reached, a third reviewer was consulted. After eliminating duplicates, the titles and abstracts were read first to exclude obvious irrelevant literature, and the full text was read further to determine final inclusion. If necessary, authors were contacted by email or telephone for unidentified if necessary, authors were contacted by email or telephone to obtain unidentified but important information relevant to the study. The extracts will include authors, year of publication, study region, basic characteristics of the study population, total number of people studied, type of study, caffeine consumption, methods of outcome assessment, outcome events, mean and SD of each scale score, and adjustment factors.

### Assessment of methodological quality

We utilized the Cochrane collaboration’s tool for assessing risk of bias to investigate the quality of the clinical trials included in this meta-analysis and JBI PACES to investigate the quality of cross-sectional trials (Higgins et al., 2011). The Cochrane collaboration’s tool for RCT trials assesses risk of bias on the following domains: selection bias, performance bias, attrition bias, reporting bias, detection bias and other bias. For each criterion, risk of bias was assessed as (1) low risk of bias, (2) unclear risk of bias, (3) high risk of bias. The JBI evaluation criteria for cross-sectional studies consisted of 10 items, each rated on a scale of 0 to 2. A score of 0 indicated that the requirements were not met; a score of 1 indicated that it was only mentioned but not described in detail; and a score of 2 indicated that a detailed, comprehensive, and correct description was given. Conflicts of opinion were discussed with a third review author until consensus is reached.

### Statistical analysis

The meta-analysis was performed by using Revman 5.4 and Stata version 12.0 software. Since all outcome indicators in this study were continuous variables and the scoring tools of anxiety scale were different, data were synthesized by using the standardized mean difference (SMD) with 95% confidence interval (CIs). For continuous outcomes, SMD with 95%CIs were calculated as the difference in means between groups divided by the pooled standard deviation (Cohen, 1988; Higgins and Green, 2010). Effect sizes with a value of *p* < 0.05 were considered significant. We utilized Dixon’s *Q*-test and the *I*-squared (*I*^2^) statistical tests to assess result heterogeneity. If there was no heterogeneity among the results (*p* > 0.1, *I*^2^ < 50%), the fixed-effect model was used for analysis. If heterogeneity existed (*p* ≤ 0.1, *I*^2^ > 50%), random effects model was used for analysis, parallel subgroup analysis or sensitivity analysis (Higgins et al., 2011). *p* < 0.05 was considered a statistically significant difference. We utilized sensitivity analysis to examine the stability of the results by removing individual trials to determine whether the removed study had a particular impact. We created funnel plots and visually examined the signs of asymmetry to investigate publication bias. Where data was missing, we contacted the authors to request further information. If data could not be obtained, we did not include the study in the meta-analysis.

Cohen’s categories were used to evaluate the magnitude of the overall effect size with (1) SMD = 0.2–0.5: small; (2) SMD = 0.5–0.8: medium, and (3) SMD > 0.8: large effect sizes (Cohen, 1988).

## Results

### Literature search and screening results

**Figure 1:**
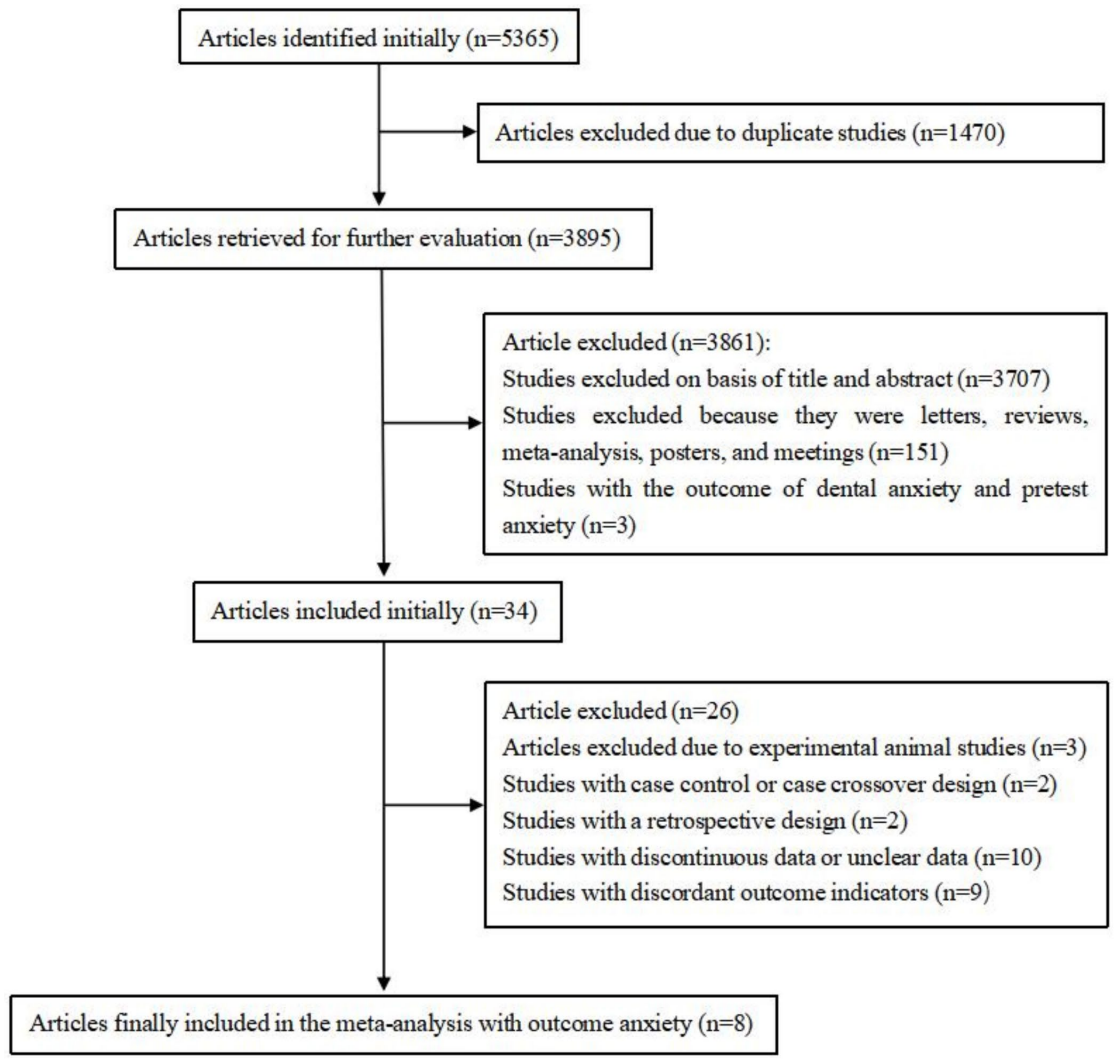
Flow diagram of included and excluded studies.

Our initial search identified 5,365 relevant articles, with 3,895 remaining after duplication. After screening titles and abstracts and excluding studies which were letters, reviews, meta-analysis, posters, and meetings, we identified 34 studies for further evaluation. Of the 34 initially included studies, we excluded 3 studies due to experimental animal studies, 2 studies with case control or case crossover design, 2 studies with a retrospective design, 10 studies with discontinuous data or unclear data and 9 studies with discordant outcome indicators. Eight studies remained in the meta-analysis (Figure 1).

### Basic characteristics of the included studies

**Figure 2:**
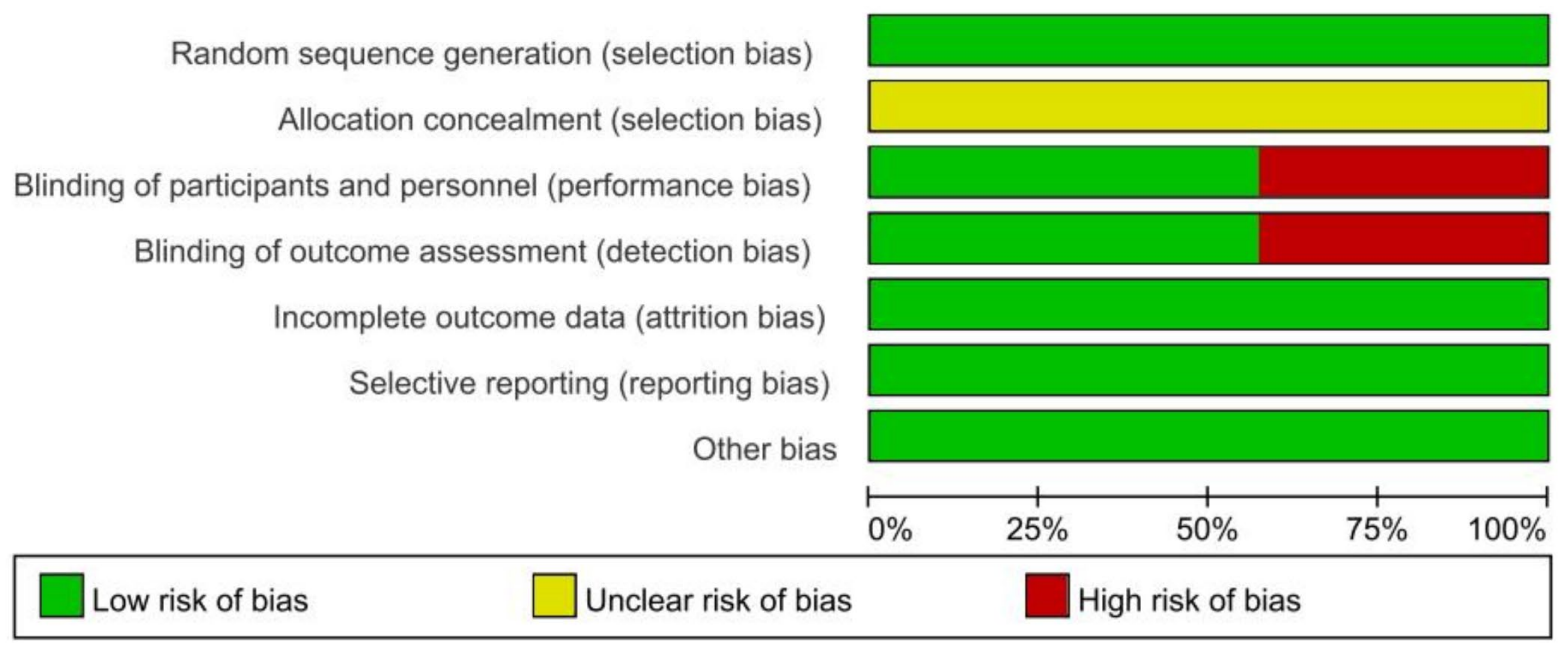
Methodological quality of included studies.

**Figure 3:**
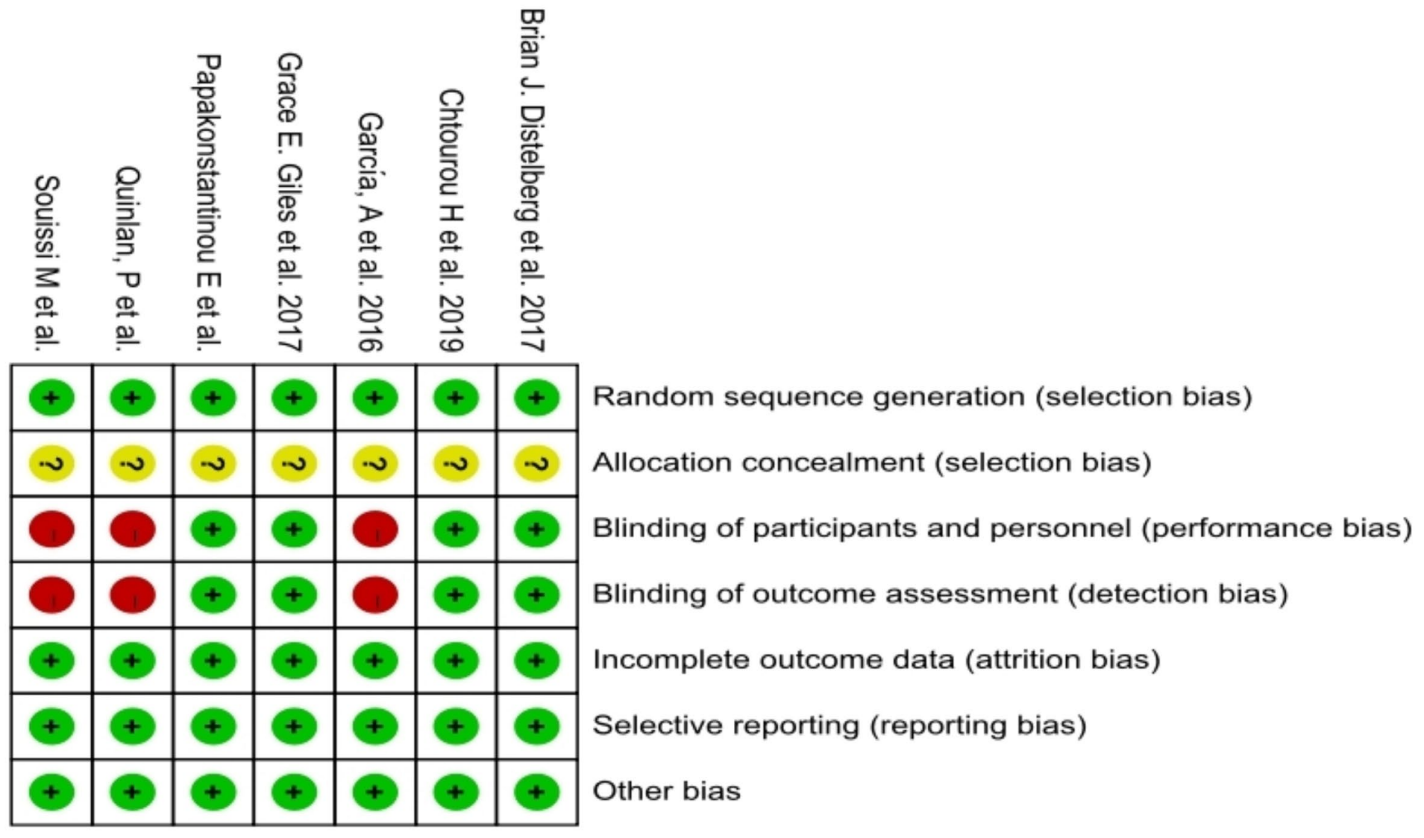
The distribution of the methodological quality of included studies.

A total of eight papers (Quinlan et al., 1997; Souissi et al., 2012; Jin et al., 2016; Papakonstantinou et al., 2016; Distelberg et al., 2017; García et al., 2017; Giles et al., 2017; Chtourou et al., 2019) were included in this study. The included studies comprised approximately 546 study participants. Characteristics of these eight studies are shown in Table 1, including seven RCTS and one cross-sectional study. Regarding the study region, two studies were conducted in Europe, two in Tunisia, three in America and one in Korea. Eight studies included adults with elevated levels of anxiety by BAI, POMS, STAI, SAS scales. According to the methodological assessment of the included literature, seven RCTS reached a moderate risk of bias (Figures 2, 3). One CS scored 16 with a high quality.

**Table 1:** Descriptive information for studies included in the meta-analysis.

Study	Study design	Sample size	Intervention/Exposure	Time of caffeine intake before the experimental session	Outcome assessment
Distelberg et al. (2017) the United States	RCT	49 Healthy participants	710 mL of either regular coffee (containing 450 mg caffeine) or decaffeinated coffee	5 day	BAI
Chtourou et al. (2019) Tunisia	RCT	19 Male physical-education students	Drinking 2 cans of “Red Bull’ beverage (containing 160 mg caffeine) or drinking a placebo	1 h	POMS
García et al. (2017) Colombia	RCT	80 Medical students	The intake of 460 mL of either an energy drink or carbonated water	1 h	STAI
Giles et al. (2017) the United States	RCT	96 Adults	Consuming 400 mg caffeine or placebo	45 or 75 min	STAI
Jin et al. (2016) Korea	CS	234 Middle school students	Daily coffee intake >27.5 mg or <4 mg	1 month	BAI
Papakonstantinou et al. (2016) Greece	RCT	40 Healthy individuals	Randomly consumed four 200 mL coffee beverages containing 160 mg caffeine	1 h	SAS
Quinlan et al. (1997) the United Kingdom	RCT	16 Habitual caffeine consumers	Subjects ingested caffeine dose or placebo	1 h	STAI
Souissi et al. (2012) Tunisia	RCT	12 Elite judoists	Beverages were ingested with/without 100 mg caffeine	1 h	POMS

BAI, beck anxiety inventory; POMS, profile of mood states; STAI, state-trait anxiety inventory; SAS, self-rating anxiety scale.

## Results of meta-analysis

### Analysis of overall effects

**Figure 4:**
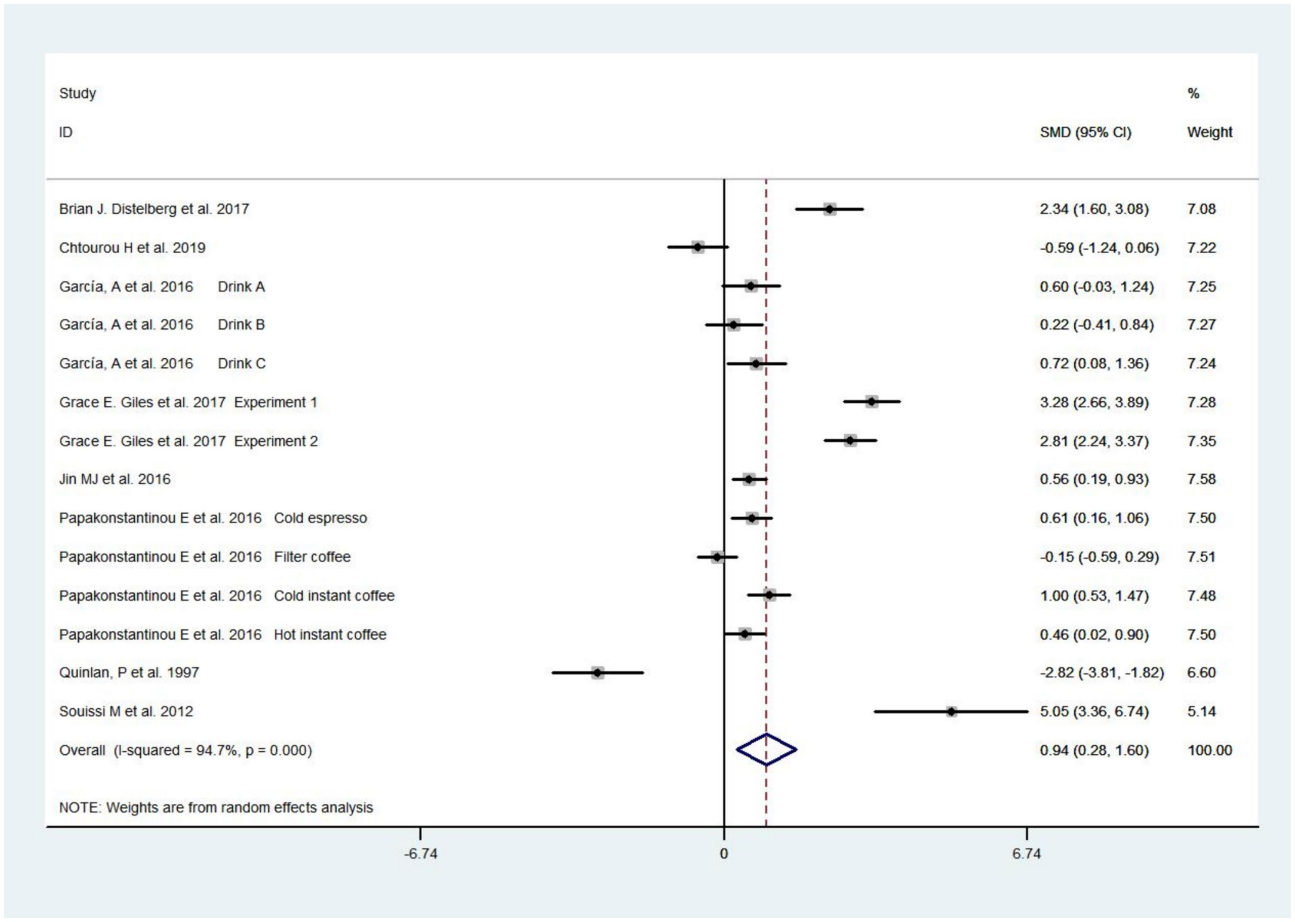
Forest plots of caffeine intake on anxiety.

Eight studies (Quinlan et al., 1997; Souissi et al., 2012; Jin et al., 2016; Papakonstantinou et al., 2016; Distelberg et al., 2017; García et al., 2017; Giles et al., 2017; Chtourou et al., 2019), including 546 people, were selected to evaluate the relationship between caffeine intake and anxiety. Meta-analysis of random effects models revealed that caffeine consumption significantly increased the risk of anxiety compared with the control group [SMD = 0.94, 95%Cl = (0.28, 1.60), *p* < 0.05; heterogeneity: *I*^2^ = 94.7% >50%, *p* < 0.001] (Figure 4). Based on Cohen’s categories, these effects were of large size.

### Sensitivity analysis and subgroup analysis

**Figure 5:**
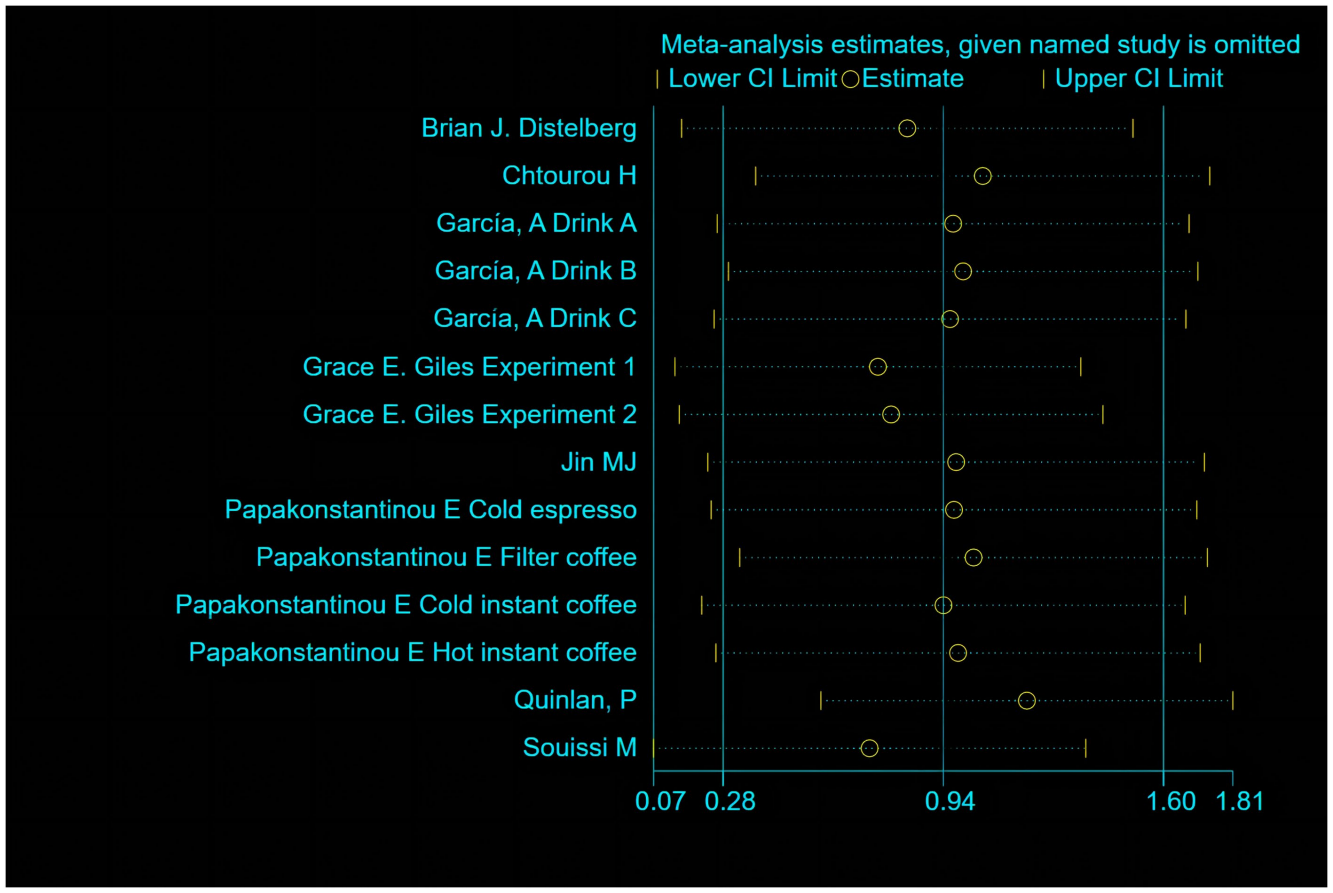
Sensitivity analysis of 8 literature.

**Figure 6:**
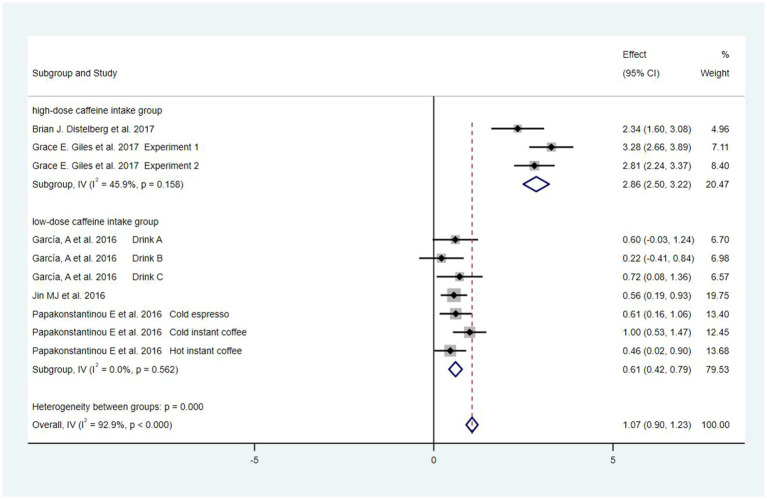
Forest plots of subgroup analysis.

Sensitivity analysis demonstrated that the literature exhibits different sensitivity profiles based on caffeine consumption (Figure 5). Subgroup analysis was performed based on coffee consumption divided into a high-dose caffeine intake group (≥400 mg) and a low-dose caffeine intake group (<400 mg), while the FDA recommended daily coffee intake was also <400 mg. By continuing the sensitivity analysis in an exclusion-by-exclusion manner, it was found that when (Souissi et al., 2012) was excluded from the high-dose caffeine intake group and (Papakonstantinou et al., 2016) filter coffee, (Quinlan et al., 1997; Chtourou et al., 2019) were excluded from the low-dose caffeine intake group, there was no heterogeneity in subgroup analysis. The results of the subgroup analysis of the fixed effects model showed a moderate increase in anxiety scores in the test group in the low-dose subgroup [SMD = 0.61, 95%Cl = (0.42, 0.79), *p* < 0.05; heterogeneity: *I*^2^ = 0% <50, *p* > 0.1]. In the high-dose subgroup, the anxiety scores of the test group increased extremely significantly [SMD = 2.86, 95%Cl = (2.50, 3.22), *p* < 0.05; heterogeneity: *I*^2^ = 45.9% <50, *p* > 0.1]. Detailed information can be found in Figure 6.

### Publication bias assessment

**Figure 7:**
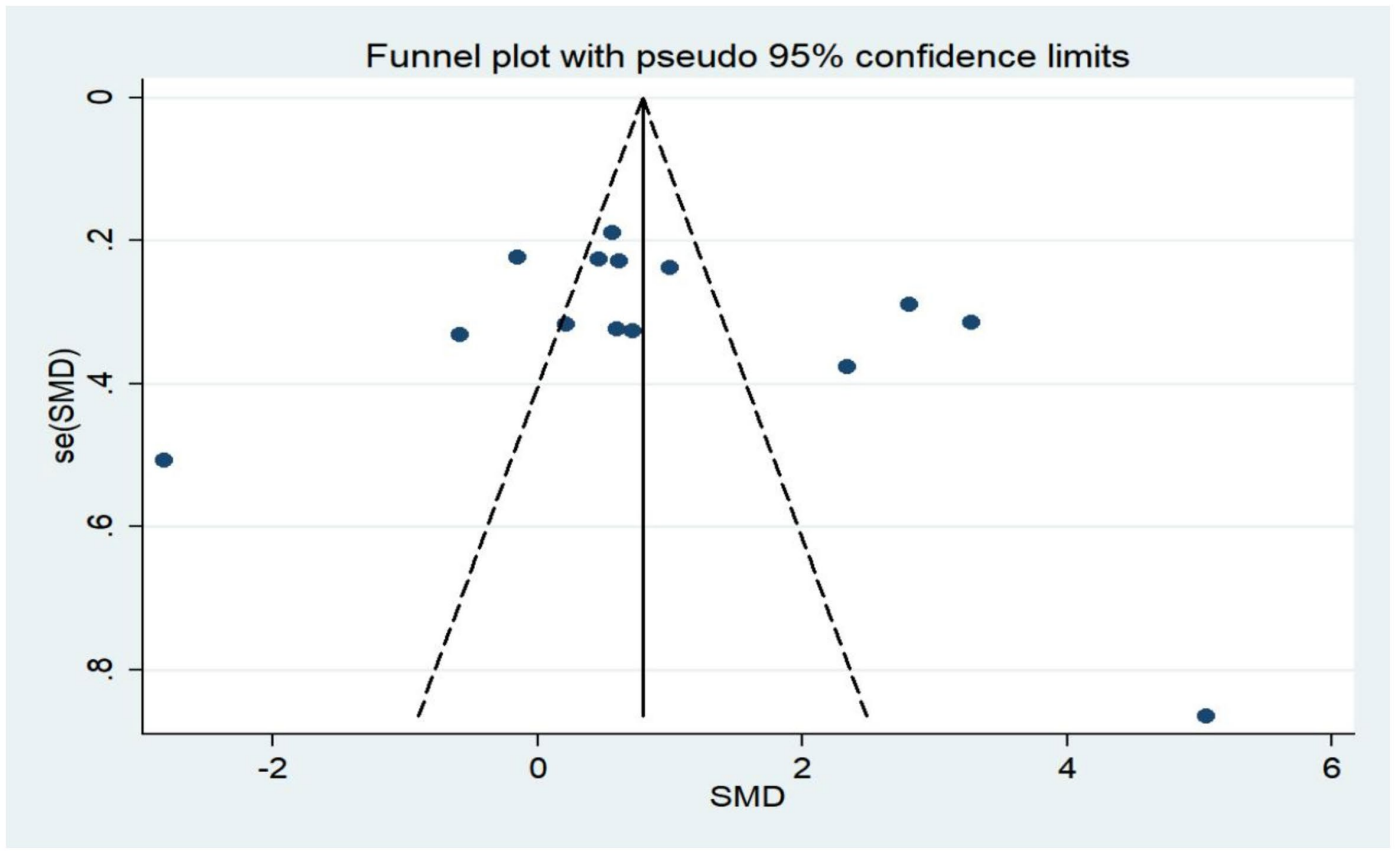
Funnel plot of caffeine intake on anxiety.

The funnel plot of this study is basically symmetrical and it can be judged that there is no publication bias in the literature of this study (Figure 7).

## Discussion

Our study was the meta-analysis of examining the association between caffeine intake and anxiety. This meta-analysis included 546 participants for caffeine intake and identified that caffeine intake was significantly associated with increased risk of anxiety in healthy people. Because of differences in baseline data collection at the time of the meta-analysis, heterogeneity between studies can easily occur, so we performed subgroup analyses based on the results of the sensitivity analyses, which showed that the high-dose caffeine (≥400 mg) group had a significantly higher increased risk of anxiety than the low-dose caffeine (<400 mg) group [SMD = 2.86, 95% Cl = (2.50, 3.22), *p* < 0.05]. Overall, this is also consistent with a number of findings from other literature (Chait, 1992; Kaplan et al., 1997) and, combined with quantitative analyses, suggests an anxietogenic effect of caffeine in healthy populations. It is noteworthy that polymorphisms of the A2A receptor influence the susceptibility of individuals to caffeine-induced anxiety (Alsene et al., 2003; Childs et al., 2008) but none of the included literature investigated A2A receptor polymorphisms, which is a potential factor for generating heterogeneity and may lead to biased results.

In discussing the clinical significance of the findings, we wondered if there was a dose–response relationship between caffeine and anxiety, but due to the small amount of literature using doses of caffeine above 460 mg, the dose–response relationship could not be analyzed. Caffeine is a central nervous system stimulant and acts in the brain through adenosine receptors, influencing attention, alertness, and producing anxiogenic effects, which may cause anxiety disorders (Aniţei et al., 2011). It also interacts with dopaminergic transmission, which is considered a different interaction from other psychostimulants such as cocaine and amphetamines (Fredholm et al., 1999). Important factors to consider clinically are that caffeine interacts with treatment and the fact that caffeine is addictive and caffeine withdrawal has been added as a diagnostic category to the DSM-5 (Hasin et al., 2013). We recommend that clinicians consider the potential anxietogenic effects of caffeine in the treatment of psychiatric disorders, and that caffeine consumption be appropriately assessed for a more individualized treatment strategy. In addition, caffeine can produce anxiety or exacerbate anxiety in adults with pre-existing anxiety disorders (Nehlig, 2016); however, the doses associated with these effects are large (1–2 g of caffeine/day) and may be consumed by only a small percentage of caffeine consumers. It has been suggested that those who experience anxiety effects from caffeine may avoid the substance (James, 1991), and that the self-limiting nature of caffeine intake would reduce any likelihood of caffeine producing anxiety in adults (Nehlig, 2016), and therefore we recommend that caffeine intake in healthy populations does not exceed 400 mg per day.

The mechanism of the association between coffee or caffeine and anxiety was still not fully established. There have been several possible biological explanations so far: First of all, caffeine is a xanthine with effects such as GABA inhibition, regulation of phosphodiesterases, activation of ryanodine receptors and non-selectively block adenosine receptors (Fredholm et al., 2005; Childs et al., 2008; Bodenmann et al., 2012), with the inhibitory A1 and predominantly excitatory A2A receptors being the most notable; the latter effects are most related to the systemic and local effects of caffeine, such as increased alertness, decreased libido, wakefulness despite sleep deprivation, irritability, fatigue, headache, seizures, weakness, and sleep and mood disorders (Aniţei et al., 2011). In this context, it is of interest that these receptors are involved in ground emotional processing. Adenosine A1 and A2A receptors are involved in the regulation of myocardial oxygen function and coronary blood flow, and antagonizing these receptors leads to increased heart rate. Psychologically, an increased heart rate may cause the body to believe that a disaster is imminent (Clark et al., 1997), meaning that the antagonistic effect caused by caffeine may cause anxiety, especially at higher doses, inducing more anxiety. Caffeine is a stimulant that affects the central nervous system, so consuming caffeinated beverages, such as coffee and tea, stimulates the central nervous system causing the body to produce and release adrenaline. This can cause a person to feel anxious or nervous (Nehlig et al., 1992). In addition, there has been no link between caffeine-induced anxiety and changes in brain activity and connectivity in healthy individuals, which could provide information on the mechanism of caffeine’s anxiolytic effects.

Our study has some limitations. (1) Due to the limited amount of included literature, there was high between-study heterogeneity in the relationship between caffeine intake and anxiety, which was eliminated by excluding individual literature in the subgroup analysis. (2) Lack of consideration of confounding factors, and although we have excluded people with other psychiatric disorders and the presence of psychotropic drug interventions, factors such as gender were not controlled for, and there are studies suggesting that caffeine increases anxiety in males but not in females (Trapp et al., 2014; Richards and Smith, 2015; Kaur et al., 2020), which may bias our results. (3) The limited range of caffeine used [0–460 mg] prevented any meaningful analysis of the dose–response relationship. (4) Our results may also be confused by side effects.

## Conclusion

In summary, the results of our meta-analysis suggest that caffeine consumption may have a detrimental effect on anxiety and may increase the risk of anxiety. This association was more pronounced at caffeine intake doses above 400 mg. Future studies should further elucidate the mechanisms of action between caffeine and anxiety from genetic risk polymorphisms to risk phenotypes. In addition, studies using a wider range of doses should be conducted to elucidate the dose–response relationship between caffeine and anxiety.

## Data availability statement

The original contributions presented in the study are included in the article/Supplementary material, further inquiries can be directed to the corresponding author.

## Author contributions

CL: Methodology, Resources, Validation, Writing – original draft, Writing – review & editing. LW: Data curation, Software, Writing – original draft, Writing – review & editing. CZ: Data curation, Writing – review & editing. ZH: Software, Writing – review & editing. JT: Data curation, Writing – review & editing. JX: Data curation, Writing – review & editing. WL: Writing – review & editing, Supervision.
